# Haemorrhage, hypercoagulability and ischaemia: Evolution of brain injury after aneurysmal subarachnoid haemorrhage

**DOI:** 10.1177/0271678X261474706

**Published:** 2026-07-24

**Authors:** Yuyang Wang, Jordan Fisker, Lipi Mishra, Matthew H Anstey

**Affiliations:** 1 Faculty of Medicine Dentistry and Health Sciences, University of Western Australia, Perth, WA, Australia; 2 Intensive Care Unit, Sir Charles Gairdner Hospital, Nedlands, WA, Australia

**Keywords:** aSAH, hypercoagulability, neutrophil extracellular trap, neuroinflammation, spreading depolarisation

## Abstract

Aneurysmal subarachnoid haemorrhage (aSAH) is a catastrophic cerebrovascular event associated with high early mortality and substantial long-term disability. Delayed cerebral ischaemia (DCI) is among its most severe complications and a principal driver of secondary neurological injury. Despite decades of research describing diverse pathophysiological mechanisms, a unifying framework for DCI pathogenesis is still lacking, and its clinical prediction remains challenging. We therefore conducted a narrative review to synthesise current understanding of the spatiotemporal evolution of brain injury following aSAH, with emphasis on integrating mechanisms across overlapping clinical phases. Acute phase events from physical effects of bleeding, including intracranial pressure elevation, hyperacute vasospasm and microvascular thrombosis, sets off the evolution of downstream brain injuries. During the early phase, converging mechanisms such as neuroinflammation, oxidative stress, spreading depolarisation and blood–brain barrier disruption coalesce into pan-vascular dysfunction and systemic hypercoagulability. Rather than representing a singular vasospastic complication, these processes collectively evolve into pathobiological states characterised by large-artery vasospasm, microvascular constriction, diffuse capillary occlusion and supply–demand mismatch, ultimately precipitating DCI and cortical infarction. This review examines the key mechanisms underlying these aforementioned processes and aims to establish mechanistic continuity across the evolving phases of injury following aSAH.

## Graphical abstract

**Graphical Abstract:**
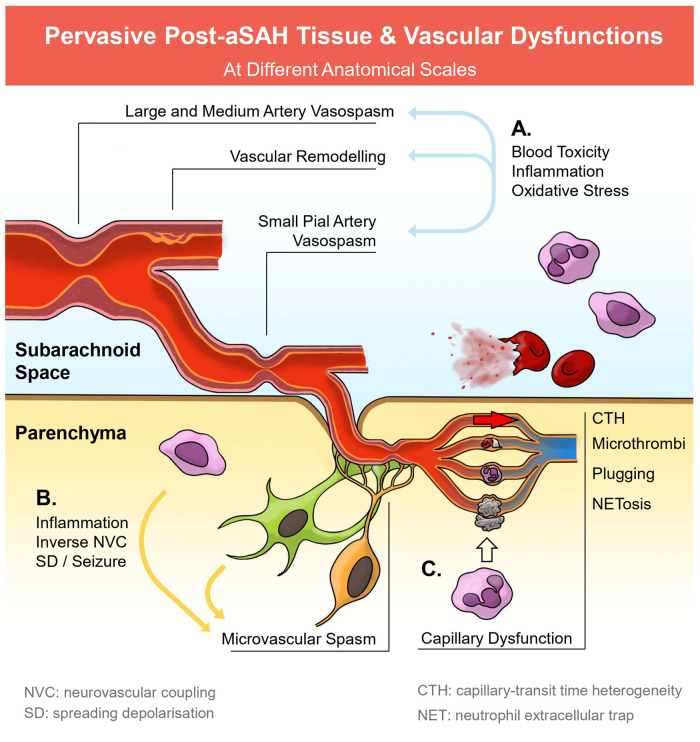
This is a visual representation of the abstract.

## Introduction

Aneurysmal subarachnoid haemorrhage (aSAH) is the catastrophic rupture of cerebral aneurysm, causing arterial bleeding into the space between the leptomeninges. Rapid extravasation of blood in the cranial vault can lead to acute cerebral ischaemia and sudden death in a non-trivial proportion of cases.^1^ Even if the brain survives the initial insult, subsequent reactive changes of damaged tissue and focal cerebral ischaemia risk further expansion of injured territories. Although aSAH historically has been regarded as a haemorrhagic stroke subtype, advances in pathophysiological understanding now implicate it as a distinct entity of ischaemic–haemorrhagic stroke.^2^ Regardless of classification, aSAH is deservedly one of the deadliest and most debilitating stroke subtype, with a pre-hospital mortality rate reaching 26% (Finnish national register)^3^; while in admission, another 13%–30% of the patients succumb to early complications and delayed brain injuries within 30 days (Danish single-centre audit)^4^; up to a quarter of the survivors live with significant disabilities post-discharge (Swiss single-centre audit).^5^

Current understanding of aSAH-associated brain injuries identifies three principal phases post-aneurysmal rupture.^6^ The initial acute phase injury occurs within 24 h post-bleed due to the physical effects of blood pooling in the cranial vault, which involve spiking intracranial pressure (ICP), reduced cerebral perfusion pressure (CPP) and acute global cerebral ischaemia.^6^ The early phase encompasses the first 72 h; whereby early brain injuries (EBI) arise from haemolysis of extravasated blood and compensatory brain changes. Mutually reinforcing pathological processes such as brain swelling, neuroinflammation, microvascular thrombosis and spreading depolarisation (SD) are the hallmarks of EBI.^7^ The delayed phase starts from 3 days onwards, at which point delayed cerebral ischaemia (DCI) evolves from EBI and cumulative effects of macro and microvascular dysfunctions.^7^ It is worth noting that pathological events under different phases overlap significantly due to the variable progression of aSAH, and that DCI may occur at any time from day 1 to several weeks post-haemorrhage, though peak incidence generally lies between days 4 and 10.^8^

**Figure 1.**
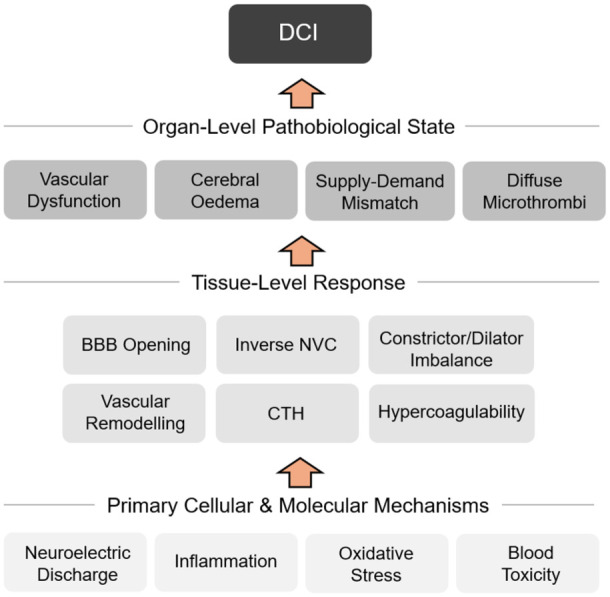
Pathophysiological mechanisms at different levels of organisation in the post-haemorrhagic brain. At the cellular and molecular level, early transient global ischaemia and blood-derived toxicity trigger neuroelectric disturbances (spreading depolarisation and seizures), neuroinflammation and oxidative stress. These mechanisms converge at the tissue level to drive blood–brain barrier disruption, constrictor–dilator imbalance, NVC, vascular remodelling, increased CTH and systemic hypercoagulability. The integration of these tissue-level responses gives rise to organ-level pathobiological states, including widespread vascular dysfunction, cerebral oedema, diffuse microthrombosis and supply–demand mismatch – collectively contributing to the development of cerebral ischaemia and infarction. CTH: capillary transit time heterogeneity; NVC: inverse neurovascular coupling.

In this review, we aim to provide an overview of the key established and emerging mechanisms of post-aSAH neurological injuries, focussing on their temporal evolution and interactions throughout the course of aSAH. We structure the discussion by first identifying acute phase events that set off the injurious cascades post-aSAH; we then explore the spatiotemporal evolution of EBI process that eventuate in DCI. To this end, we distinguish tissue-level emergent pathobiological states (e.g. vasospasm, microthrombosis, cerebral oedema) from the primary contributing mechanisms at the cellular and molecular level (e.g. neuroinflammation, oxidative stress, spreading depolarisation), to reflect the complex multifactorial origin of post-aSAH injuries (Figure 1).

## Injury cascades start in the acute phase

Acute-phase brain injuries arise from rapid global and focal cerebral haemodynamic disruptions that occur immediately after aneurysmal rupture.^6^ In the first moments to the next 15–30 min, transient ICP spike, hyperacute cerebral vasoconstriction and platelet aggregation are the three major processes that mediate acute ischaemia. Given that timely neurosurgical/endovascular aneurysm securement drastically reduces lethal complications, peri-onset hyperacute brain injuries and downstream peripheral organ dysfunctions before intervention now constitute major sources of aSAH mortality.^9^ Acute-phase events also lay the foundation for the subsequent evolution of early and delayed phase injuries, yet the area remains comparatively understudied. Unfortunately, clinical investigation of the peri-onset acute phase is challenging partly due to the timing of patient presentation, and much of our current understanding is therefore derived from animal models.

### Intracranial pressure dynamics and global cerebral ischaemia

Transient intracranial hypertension develops abruptly as the physical effect of arterial blood injection into a volume-limited cranial vault, which rapidly overwhelms compensatory mechanisms like intracranial volume buffering and cerebral autoregulation.^10,11^ Immediately afterwards, CPP falls proportionally given the largely unchanging mean arterial pressure (MAP). This acute reduction in perfusion pressure may benefit haemostasis at the rupture site, but nonetheless can lead to the onset of acute global cerebral ischaemia.^10^ If present, such brief but intense global perfusion deficits clinically manifest as loss of consciousness at presentation, which is observed in a substantial proportion of patients and is associated with poorer clinical grade at presentation, greater haemorrhage severity on the modified Fisher scale (mFisher), and independently predicts adverse functional outcomes at 12 months as measured by the modified Rankin Scale (mRS).^12^ The severity of acute global ischaemia in these first moments is likely determined by the magnitude of the ICP spike; in contrast, brain injury from elevated ICP in later phases reflects more complex interactions between pressure magnitude, cumulative burden and variability.^13–15^ To put into perspective, the initial transient ICP spike can approach or exceed MAP, resulting in a peak pressure, that is, many folds greater than elevated ICP values in the early and delayed phase (e.g. 80–160 vs 20–30 mmHg).^16–18^ The magnitude of this initial peak pressure, in turn, is dependent on both the volume and the velocity of extravasation during active bleeding.^11^ In other words, greater peak pressure may arise from either a large volume haematoma or a high-velocity jet of blood, but is most likely determined by the interaction of both.

Across the entire course of aSAH, pathological rise of ICP likely follows at least three distinct phases, each governed by different mechanisms. First, before haemostasis, ICP rises abruptly, with its magnitude dictated by haemorrhage volume, velocity and the resulting pressure dynamics. Following haemostasis, ICP either declines and stabilises within minutes thanks to intracranial volume buffering, or becomes persistently elevated if buffering mechanisms like CSF outflow fail.^19^ In both cases the residual pressure is largely determined by existing haematoma volume. Finally, during the early and delayed phases, a moderate but sustained rise of ICP may occur, its trajectory would then be additionally driven by the evolving secondary injury processes or intracranial complications, such as cerebral oedema and hydrocephalus.^20^ At this stage, poorer functional outcome as measured by the Extended Glasgow Outcome Scale (GOSE) often follows long cumulative duration of ICP elevation above a critical threshold (typically 20 mmHg).^21^ It is worth noting that ICP is normal in a significant proportion of patients at presentation, likely because the initial transient surge has already subsided by the time of measurement.^22^ Nevertheless, in the acute phase, cerebral blood flow (CBF) reduction does not always resolve after ICP/CPP normalisation, suggesting other processes at play.^19^

### Hyperacute vasospasm augment acute cerebral ischaemia

The earliest cerebrovascular response post-rupture is an intense localised vasoconstriction (i.e. hyperacute vasospasm) of both large arteries and small pial arterioles within the blood contaminated territories, which constitutes a second major cause of acute ischaemia.^23^ Hyperacute vasospasm occurs within minutes following aneurysmal rupture and self-resolves within 20 min in human and primate models.^23^ The underlying mechanism of this vascular phenomenon is currently unknown and remains understudied; nevertheless, promising theoretical causes include mechanical disturbance facilitated by subarachnoid anatomy and acute neurogenic injury responses.

The potential mechanical cause of hyperacute vasospasm is the mass effect of developing haematomas, which may become amplified by the anatomical properties of the subarachnoid space.^23^ The basal subarachnoid space is interrupted by dense non-perforating trabecular arachnoid membranes (e.g. Liliequist’s membrane) that may limit the even distribution of extravasated blood.^24^ An evolving haematoma and the surrounding CSF may thus become briefly compartmentalised and compress local vasculature and the closely associated trabecular fibres, leading to short-lived vasospasm that dissipates when the haematoma stabilises.^24,25^ Still, direct experimental evidence supporting this theory is lacking.

Activation of the central sympathetic nervous system after aSAH is a potential neurogenic cause of hyperacute vasospasm, as well as the characteristic acute peripheral organ dysfunctions such as neurogenic pulmonary oedema and cardiac arrhythmia.^26^ The pathway of central sympathetic activation post-aneurysmal rupture is not fully understood, but likely involves pain response and global cerebral ischaemia from the ICP spike, which impairs extracellular glutamate reuptake, leading to excitation of the hypothalamic paraventricular nucleus and rostral ventrolateral medulla (RVLM). It also remains unclear whether this sympathetic-driven vasoconstriction occurs at the level of cerebral arteries or arterioles. Hyperacute sympathetic-driven vasospasm of large and medium arteries was observed in animal models.^27,28^ However, this has not been reliably demonstrated in humans, where acute CBF disruption attributable to sympathetic activity appears to occur at the level of arterioles and below.^29,30^ Such discrepancy may be the result of interspecies differences in adrenergic receptor distribution. Nevertheless, the number of clinical studies on this topic is limited. Regardless of the vessel calibre, the magnitude of the acute phase sympathetic overdrive is such that cerebral blood vessels in animal models develop a pseudo-denervation sensitivity lasting around 7 days, and thus become primed for subsequent delayed vasospasm.^26^ This potential priming mechanism is supported by an increased responsiveness of post-aSAH human cerebral arteries to α1-agonists in an autopsy report.^31^

### Microvascular thrombosis starts off in the acute phase

The concurrent ICP spike and hyperacute vasospasm can lead to rapid onset of devastating global cerebral perfusion deficits. One of the earliest consequences of this acute ischaemia is endothelial injury, which precipitates intravascular platelet aggregation and thrombosis.^32,33^ Indeed, the acute no-reflow phenomenon has been characterised in animal models, where the initial CBF deficit persists even with complete patency of larger vessels and normalisation of CPP, most likely due to microcirculatory dysfunction partly driven by occlusion of capillaries, small arterioles and venules.^34,35^

Two distinct but related types of thrombotic events may occur in the acute phase of aSAH.^36^ The first type is microvascular platelet plug that develops rapidly but is largely reversible. The formation of platelet plugs follows a bimodal temporal pattern, first peaking at 10 min post-rupture, followed by a second peak at 24 h, eventually dissipating by 48 h.^36,37^ The second type constitutes true fibrin-interlinked microthrombi that develop around 3 h post-rupture but could persist for days to weeks, which are spatially well-correlated with delayed focal infarctions on autopsy studies.^38,39^ Both types of thrombotic events depend upon platelet activation, which is a physiological response to vessel rupture and injury. However, the diffuse and sustained nature of post-bleed thrombotic events reflects an early pathological platelet response that extends beyond physiological haemostasis at the rupture site.^34,37^ Apart from direct occlusion, acute platelet aggregation may additionally contribute to microvascular spasm through platelet-derived vasoactive mediators such as serotonin, adenosine diphosphate (ADP) and platelet-derived growth factor (PDGF).^40^

The mechanisms underlying such platelet response are likely multifaceted as most acute phase events are prothrombotic in nature. For example, nitric oxide (NO) scavenging likely intensifies as cell-free haemoglobin level rises in the hours following initial rupture, which not only augments acute phase vasospasm but also stimulates platelet activation through the failure of NO–cGMP–PKG-dependent inhibition.^41^ Similarly, hyperacute spasm of pial arterioles drastically increases vascular resistance, while elevated ICP compresses venous outflow – altogether leading to reduced flow and blood stasis in the capillary beds. Finally, with the consequent endothelial injury due to acute cerebral ischaemia, the proverbial Virchow’s triad is satisfied, whereby the post-haemorrhagic brain creates the ideal condition for microvascular thrombosis. There are additional mechanisms that amplify microthrombi formation, including inflammatory plate–leukocyte interactions, development of systemic hypercoagulability and neutrophil-driven thrombosis. These are most pronounced in the early to delayed phase and will be discussed in the next sections.

## The evolution from EBI to DCI

**Figure 2.**
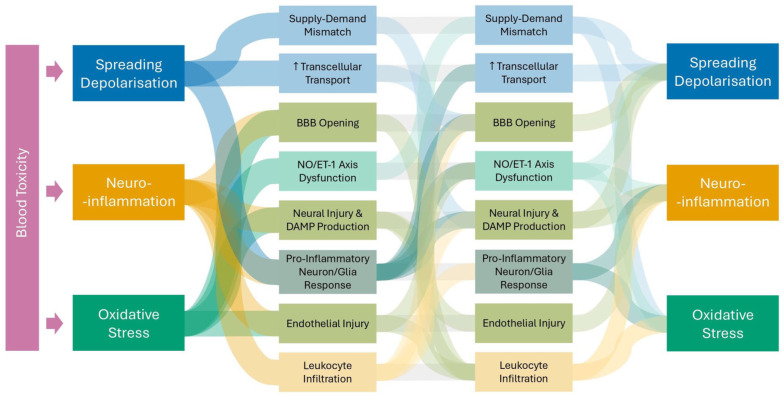
Mutually reinforcing primary cellular and molecular pathological mechanisms in aSAH. Flow chart of positive feedback loops linking each primary cellular and molecular injury mechanisms. From left to right: blood toxicity initiates spreading depolarisation, neuroinflammation and oxidative stress during the early phase. These primary cellular and molecular processes subsequently drive multiple forms of downstream tissue injury, which in turn further amplify the initiating primary mechanisms. The pathways illustrated here are not intended to be exhaustive, but rather to highlight the extensive interdependence and positive feedback between major pathological processes. BBB: blood–brain barrier; NO: nitric oxide; ET-1: endothelin-1; DAMP: damage-associated molecular pattern; SD: spreading depolarisation.

**Figure 3.**
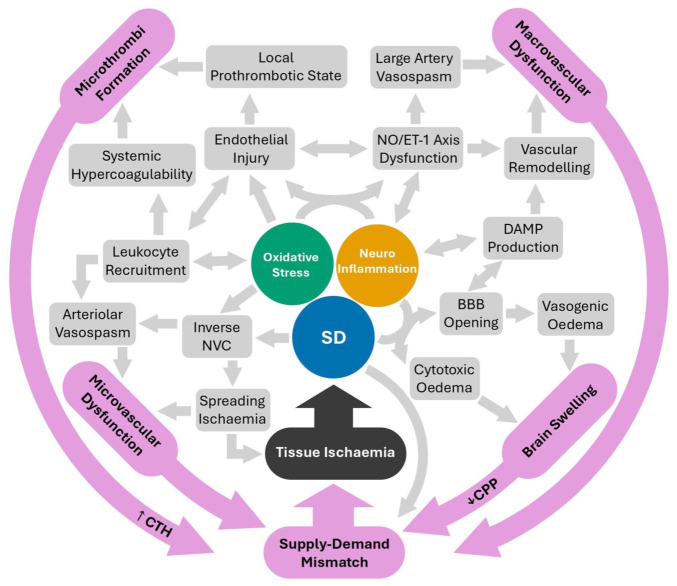
Major pathways of cellular and molecular mechanisms driving tissue and organ-level dysfunctions. Schematic overview of the major pathways linking primary injury mechanisms to tissue- and organ-level dysfunction, including micro- and macrovascular dysfunction, microthrombi formation, brain swelling and supply–demand mismatch, which converge on tissue ischaemia that further reinforces upstream pathological processes. The relationships illustrated here are not exhaustive due to limitations of 2D representations. Refer to text for details. BBB: blood–brain barrier; NO: nitric oxide; ET-1: endothelin-1; DAMP: damage-associated molecular pattern; SD: spreading depolarisation; NVC: neurovascular coupling; CTH: capillary transit time heterogeneity; CPP: cerebral perfusion pressure.

The lack of a unified and internally consistent terminology in the field necessitates clarification of the concepts under discussion. Currently, EBI has no consensus definition and generally describes the diverse injurious processes arising within the first 72 h, whereas DCI represents secondary focal cerebral ischaemia that occurs despite initial aneurysm securement, with a rigorously defined clinical manifestation.^42^ The transition from EBI to DCI is best viewed as a continuum rather than discrete events. During EBI, oxidative stress, neuroinflammation, SD, blood–brain barrier disruption and tissue injury progressively reinforce one another (Figure 2). DCI emerges when these cumulative processes result in sustained perfusion–metabolic mismatch, characterised by pan-vascular dysfunction, impaired tissue oxygen extraction, heightened neural metabolism and ultimately focal cerebral ischaemia (Figure 3).

Transient ischaemia in the acute phase tends to self-resolve within hours but initiates the injurious cascades of EBI that eventually abate or progress to focal cerebral ischaemia and infarction.^43,44^ The course of CBF recovery after the initial acute ischaemia is variable: no recovery means certain death within hours (<40% baseline in animal models), survivors may see CBF return to baseline in the first 48 h or suffer from a sustained hypoperfusion in the early phase; delayed phase CBF reduction from numerous causes may occur from day 3 onwards even in the absence of these early perfusion deficits.^45^ Nonetheless, mean transit time on early phase CT perfusion remains one of the most promising emerging predictors of DCI occurrence.^45^

In the hours to days following the initial haemorrhage event, the acute phase primary brain injuries become established.^6^ Circulatory dysfunction can become consolidated in the setting of persistent intracranial hypertension and sustained pro-vasospastic and prothrombotic microenvironments. Oxidative stress aggravates as cellular metabolism becomes disrupted and haematoma degradation begins to release oxidising Hb-derivatives. SD may grow more frequent and destructive in the increasingly metabolically challenged neural tissue. Neuroinflammations intensify in response to various forms of cellular injuries. These primary pathophysiological mechanisms evolve in parallel and eventually converge on major pathobiological states, such as brain swelling, vasospasm (at all levels of cerebral vasculature) and microthrombosis, among others. Ultimately, many mechanisms contribute to the eventual onset of DCI during the transition between early and delayed phase, but all result in either absolute ischaemia due to blood flow reductions or relative ischaemia from supply–demand mismatch.

### Brain swelling and blood–brain barrier disruption

Persistent swelling of brain tissue in the early and delayed phase can lead to global cerebral ischaemia and DCI. Conversely, cerebral ischaemia and infarction arising from other causes further perpetuate brain swelling.^46^ Accordingly, the prognostic significance of early cerebral oedema (within 72 h) is increasingly recognised, with tools such as the Subarachnoid Hemorrhage Early Brain Edema Score (SEBES) independently predicting in-hospital complications and long-term functional outcomes.^47,48^ The standalone evolution of cerebral oedema can be mechanistically divided into early (cytotoxic and ionic) and delayed (vasogenic) components that are initially driven by a combination of osmotic and electrochemical forces acting on neural cells, later augmented by fluid extravasation across an increasingly permeable blood–brain barrier (BBB).^49,50^ Nonetheless, both forms of oedemata arise from interdependent pathophysiological processes and evolve along a continuum.

Early cerebral oedema can be rapidly induced by the initial global ischaemia after aneurysmal rupture and further develops during the acute and early phase. Evidence of brain swelling can be observed in up to 67% of the initial CT scans at presentation and is detectable within minutes to hours in animal models.^50–52^ With a yet intact BBB, cytotoxic oedema occurs first under ischaemia as energy-dependent ion pumps fail, leading to neural cell swelling and shrinkage of extracellular compartments following intracellular shift of water.^46,49^ Mechanistically, early cytotoxic oedema is the downstream morphological correlate of acute spreading depolarisation (SD), which triggers an abrupt non-selective increase in membrane conductance, leading to free exchange of permeant ions and the complete collapse of transmembrane ion gradients.^46,53^ During SD, more sodium (Na^+^) and chloride (Cl^−^) ions enter the cells than potassium (K^+^) efflux, resulting in intracellular shift of water that manifests as neural cell swelling and dramatic contraction of extracellular space volume (up to 70%).^46^ Ionic oedema follows suit as CSF flows into the now small but comparatively hyperosmotic extracellular compartment through the glymphatic system, leading to macroscopic tissue swelling in the affected cortical regions.^54^ Depending on the duration and severity of perfusion deficit, SD-driven cytotoxic oedema initially remains reversible with reperfusion but becomes irreversible beyond a critical commitment point, reached within minutes to tens of minutes depending on residual perfusion.^46^ Past the commitment point, persistently depolarised cells commit to dying and the affected tissue becomes necrotic (electrographically as the negative ultraslow potential (NUP) on electrocorticography), driving downstream neuroinflammation and reinforcing the vasogenic delayed cerebral oedema.

The underlying processes involved in delayed cerebral oedema kick off simultaneously with early oedema but manifest slowly over hours to days.^49,51,55^ Late brain swelling involves an additional vasogenic component, as the increasing BBB permeability permits paracellular diffusion of ions, proteins and fluid from plasma into extracellular space, driven principally by hydrostatic pressure.^50^ A leaky barrier also disrupts the normally tightly regulated extracellular ionic environment, which may further exacerbate cytotoxic oedema by increasing the propensity of SD.^56^ Neuroinflammation is likely among the most important causes of BBB opening.^57^ Inflammatory mediators such as proteases, growth factors and reactive oxygen and nitrogen species can synergistically degrade extracellular matrices (ECM) critical for barrier functions.^58^ The identity of these effectors highlights the contradicting physiological needs between maintaining barrier integrity versus vascular remodelling and repair. For example, matrix metalloproteinase-9 (MMP-9) is produced in both intravascular and cerebral compartments by platelets, endothelial cells, neutrophils and reactive glia, where it degrades endothelial tight junctions as well as basement membrane components, including collagen IV and laminin, eventually allowing new ECM to be laid down.^59–61^ Similarly, vascular endothelial growth factor (VEGF) secreted by injured endothelial cells, pericytes and astrocytes down-regulates tight junction protein expression to facilitate vascular repair and reorganisation at the expense of BBB integrity. Reactive oxygen and nitrogen species (ROS and RNS) produced by inflammatory cells and Hb derivatives directly damage junctional and basement membrane proteins, while simultaneously amplifying inflammatory responses in a positive feedback loop.^33,62^ Altogether, the breakdown of barrier-forming junctions and ECM, prior to repair, promotes cerebral oedema by increasing paracellular diffusion of plasma contents into the extracellular space.

Neuroinflammation may also increase barrier permeability by upregulating transcellular transport across the BBB. An emerging link between inflammation and brain swelling is the sulfonylurea receptor 1 – Ca^2+^ activated transient receptor potential melastatin 4 (SUR1–TRPM4) complex – a non-selective monovalent cation channel that becomes acutely upregulated in response to neuroinflammation, in virtually all cell types of the neurovascular unit.^63,64^ Increased expression of endothelial SUR1–TRPM4 channels may directly drive influx of Na^+^ and water across the BBB. Occurrence of repetitive SD in the early phase also reciprocally strengthens inflammation and barrier breakdown, by both enhancing glia-derived inflammatory signals through activation of intracellular calcium waves, and stimulating caveolin-1-dependent transcytosis across the endothelium.^65,66^ Overall, heightened transcellular and paracellular transports due to inflammation and SD act in concert to drive the progression of vasogenic delayed cerebral oedema. Importantly, these mechanisms do not act independently. Prolonged cell swelling and lysis initiate tissue inflammation. Neuroinflammation then promotes oxidative stress and BBB disruption, while BBB breakdown facilitates further inflammatory cell recruitment and SD ignition, completing the cycle (Figure 2).

### Vasospasms reflect convergent injury mechanisms

**Figure 4.**
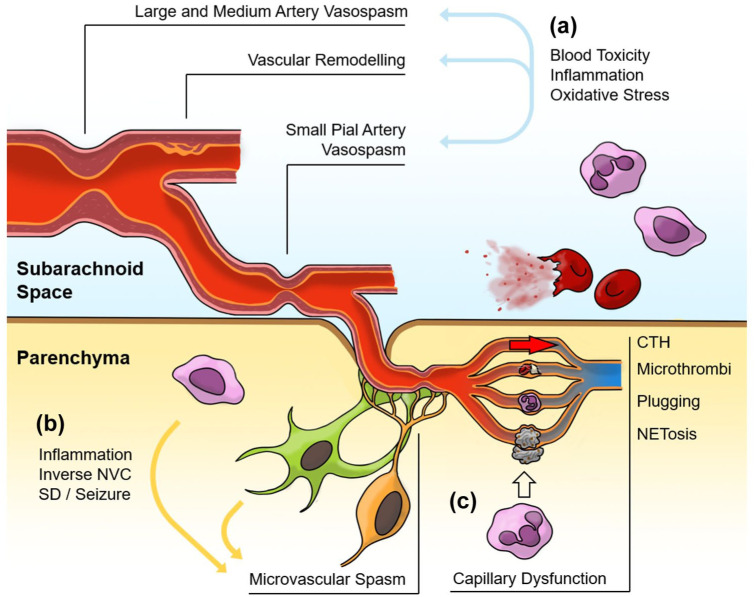
Vascular dysfunction across vessel calibres following aneurysmal subarachnoid haemorrhage: (a) large- and medium-sized artery vasospasm arises in the subarachnoid space in response to blood toxicity, inflammation and oxidative stress, with additional contribution from vascular remodelling. Small surface pial arteries are similarly affected, (b) at the level of penetrating arterioles, microvascular spasm is driven by parenchymal processes, including neuroinflammation and inversion of NVC. SD and electrographic seizures further impair perfusion by promoting spreading ischaemia and increasing metabolic demand, and (c) capillary dysfunction predominantly reflects luminal obstruction from microthrombi, NET-associated thrombosis and direct leukocyte plugging, extending from precapillary segments to postcapillary venules. Occluded branches increase CTH and further impair tissue oxygenation. CTH: capillary transit time heterogeneity; NET: neutrophil extracellular trap; NVC: neurovascular coupling; SD: spreading depolarisation.

Blood flow disruption following aSAH evolves from an initial transient global ischaemia at rupture, to a mixed global–regional perfusion deficit during EBI, and finally to predominantly focal ischaemia at the onset of DCI.^44,67^ This progression reflects increasingly localised vascular and neurovascular disruption, with the early phase representing a critical transitional period bridging acute injury and delayed ischaemia. Global perfusion deficit can result from cerebral oedema and ICP elevation that impede blood entry into the cranial vault, or diffuse capillary dysfunction due to neuroinflammation and microvascular thrombosis. In contrast, focal perfusion deficits largely arise from pathological vasoconstriction, or vasospasm, which can impact both macro and microvascular circulation. Notably, different mechanisms underlie vascular dysfunction across different vessel calibres, highlighting the difficulty of using monotherapies to improve cerebral perfusion post-aSAH (see summary Figure 4).

### NO/ET-1 axis dysfunction is central to vasospasm onset

In the classical sense, cerebral vasospasm is the sustained constriction of large and medium arteries detected angiographically, extending from internal carotid to pial arteries and causes territorial hypoperfusion.^68^ An imbalance between dilatory and constrictive vasoactive substances, in particular dysfunction in the nitric oxide/endothelin-1 (NO/ET-1) axis, is one of the major contributors to vasospasm onset, among a range of other vasoactive substances produced post-bleed.^69^ A variety of interconnected processes may reduce the availability of vasoactive NO following aneurysmal rupture, such as scavenging by cell-free Hb derivatives, nitric oxide synthase (NOS) uncoupling and consumption by myeloperoxidase derived from reactive glia and infiltrating leukocytes.^70^ However, the overall effect of aSAH on cerebral NO homeostasis is likely triphasic, with an initial acute depletion after rupture, a relatively rapid restoration to baseline levels within hours, followed by a global increase in NO production that coincides with EBI progression in the early phase.^71^ On top of scavenging and consumption, the changing NO levels and distribution are additionally driven by NOS expression and activities.^72,73^ Therefore, post-haemorrhagic NO dynamics likely reflect a complex spatiotemporal dysregulation rather than simple NO depletion alone.

The early phase marks the reduction of NO from endothelial (eNOS) and neuronal (nNOS) isoforms, which are the main sources of vasoactive NO that regulate vascular tone and neurovascular coupling.^71,72^ Both the expression and enzymatic activities of eNOS and nNOS are calcium-dependent, and their dysfunction post-aSAH presumably results from reduced Ca^2+^ influx due to inactivation of Ca^2+^ channels by Hb derivatives.^74–77^ On the other hand, excess NO is produced by the inducible isoform (iNOS), which is expressed in virtually all cell types of the neurovascular unit in response to injury.^77^ Ischaemia, neuroinflammation and oxidative stress prevalent in the post-haemorrhagic brain potently induce iNOS expression; once active, iNOS continues to produce NO independent of Ca^2+^ activation signals.^78,79^ NO dysfunction may additionally occur via both direct NOS inhibition and uncoupling, which affect all three isoforms. NOS activities can be directly inhibited by Hb derivatives such as bilirubin oxidation products (BOXs); whereas uncoupling occurs with substrate/cofactor depletion, which predispose production of superoxide radicals in place of NO.^80,81^ The key NOS cofactor tetrahydrobiopterin (BH_4_) can become inactivated by ROS following aSAH, leading to a vicious cycle of self-reinforcing oxidative stress through NOS uncoupling. Overall, reduced vascular NO (eNOS-derived) unleashes vasoconstriction, thrombosis and vascular remodelling, whereas increased parenchymal NO (iNOS-derived) sustains tissue oxidative/nitrative stresses and neuroinflammation.

The loss of the vasodilator NO permits increased production of the potent vasoconstrictor endothelin-1 (ET-1). Increased ET-1 expression begins in the acute phase in endothelial cells, as NO depletion removes the key inhibitory control, and global ischaemia enhances its expression through hypoxia-inducible factor-1α (HIF-1α) activation.^82,83^ In the early phase, neuroinflammation further increases ET-1 expression, with the dominant source shifting toward reactive astrocytes, microglia and infiltrating peripheral leukocytes, which express ET-1 in response to DAMP and proinflammatory cytokine stimulation.^57,84^ ET-1 dynamics following aSAH are less predictable and appear to be damage-dependent. Significant increase of ET-1 in CSF generally occurs at day 3 and peaks around day 7.^85–87^ Based on such pattern, ET-1 does not appear to be a dominant effector in the early phase; rather, its expression is largely shaped by EBI, which in turn drives delayed phase events including vasospasm and sustained inflammation. ET-1 exerts its vascular effects via binding endothelin A receptors (ET_A_) widely expressed in vascular smooth muscle cells and pericytes, which stimulate potent vasoconstrictions and drive pro-contractile transformations.^88,89^ ET-1–ET_A_ interactions also exert broader secondary effects such as perpetuating inflammation, oxidative stress and promoting pathological neuroelectric discharges (i.e. seizures and SD, via inhibition of glutamate reuptake).^90–93^ Collectively, ET-1-mediated vasoconstriction acts additively with other local vasospastic mediators such as serotonin, thromboxane and eicosanoids released following aSAH.^73^ Their summed effects precipitate cerebral vasospasm, worsening neuroinflammation and microvascular dysfunction.

### Vascular remodelling provides structural basis of vasospasm

Biochemical imbalance of vasoactive substances is not the sole cause of vasospasm in the delayed phase, as spasmogenic cellular and ultrastructural changes in affected arteries are observed in both autopsies and animal studies.^94,95^ The most striking morphological changes in large arteries after aSAH is the corrugation of intima and desquamation of endothelial cells.^96^ The pathological significance of such intimal morphology remains unclear, though it may contribute to a stenosed vascular lumen and hydrodynamic perturbations. This intimal morphology develops due to extensive ingrowth of granulation tissues between intima and media, leading to subendothelial fibrosis and vacuolations that detach endothelial cells from the ECM. The degree of intimal distortion appears to be damage-dependent, with greater wall changes correlating with higher haematoma volume.^97^ The muscular media layer undergoes pro-contractile modifications, marked by increased media thickness driven by smooth muscle proliferation and pro-contractile phenotypic transformation.^98,99^ The muscle architecture also becomes disarrayed as cells migrate into the subendothelium and adventitia.^100^ Finally, the adventitial layer is often laden with blood clots and cellular remnants, potentially obstructing perivascular fluid flow and exacerbating inflammatory wall damage.^101^

The overall vascular compliance decreases as the arterial wall stiffens in the delayed phase following aSAH.^102,103^ The vessel wall stiffening is potentially preceded by an initial increase in compliance due to MMP-mediated ECM degradation (days 1–3, also contributing to vasogenic oedema),^104,105^ which could gradually recover following delayed upregulation of endogenous inhibitors such as tissue inhibitor of metalloproteinase 1 (TIMP-1) from day 3 onwards.^106^ Indeed, ECM contents eventually return to baseline level by day 7 in animal models.^107,108^ In human patients, MMP-9 level rises sharply from day 1 post-bleed and persists for weeks, lagged by gradual increase in TIMP-1 level that peaks around day 9.^109^ Given that clinical biomarker trends broadly parallel animal data, a hypothetical timeline of vessel wall compliance changes would comprise a gradual increase during the first week post-bleed, followed by a return towards baseline by the end of this period, and finally a progressive stiffening from week 1 onwards. The subsequent reduction in vessel compliance results from increased deposition of collagen, fibronectin and tenascin – all requiring activation by growth factors such as VEGF and transforming growth factor β (TGF-β).^110,111^ Such growth factor signalling becomes amplified in the delayed phase as the pro-inflammatory signal wanes and pro-healing processes predominate. The remodelling at this stage may also become self-reinforcing, as increased wall stiffness stimulates mechanotransduction that further reinforces ECM deposition via yes-associated protein (YAP) dependent pathways.^112^

Nevertheless, significant knowledge gaps remain, as the interplay between arterial remodelling and vasospasm has received relatively little attention compared with other pathophysiological mechanisms in aSAH. For example, it remains controversial whether arterial wall thickening contributes to luminal narrowing during vasospasm; the dynamic evolution of wall composition from the early to delayed phase also remains to be elucidated. Most importantly, the clinical and prognostic significance of the post-bleed vascular remodelling is still unknown. A single study reported no correlation between MMP/TIMP dynamics and functional outcomes as measured by the mRS and Glasgow Outcome Score, at discharge or 6 months.^109^ However, no study has comprehensively examined the prognostic value of both morphological and biochemical changes involved in remodelling.

### Microvascular spasm occurs due to inverse neurovascular coupling

Microvascular spasms occurring at the level of arterioles and below lie beyond the detection threshold of routine angiographic techniques, yet constitute a major threat to perfusion in the early to delayed phase due to their effect on small resistance vessels (i.e. from surface pial arteries to penetrating arterioles).^36^ Indeed, early-phase CT perfusion (CTP) and digital subtraction angiography (DSA) parameters reflecting microcirculatory dysfunction predict poorer functional outcomes as measured by the mRS, at both discharge and 3-month follow-up.^113,114^ Cerebral arterioles are subject to the same driving forces of macrovascular spasm (e.g. constrictor-dilator imbalance and wall damage) but are additionally affected by a progressive disturbance in neurovascular coupling due their proximity to the neuropil in the post-haemorrhagic brain.^115^

There are likely two distinct modes of altered neurovascular coupling following aSAH.^116^ First, global ischaemia (i.e. during acute phase) may attenuate the physiological neurovascular coupling, resulting in reduced vasodilation and blunted functional hyperaemia. Unfortunately, this phenomenon is only documented in rodent models in the context of somatosensory evoked potential.^117,118^ It therefore remains unclear whether such flow attenuation occurs in humans during the transient global ischaemia following aneurysmal rupture, and whether it persists in the otherwise well-perfused tissue in the early and delayed phase. In contrast, the second mode of neurovascular disturbance constitutes an inversion of normal hyperaemic response, whereby increased neural activity results in sustained vasoconstriction instead of dilation. The inverse neurovascular coupling is most pronounced in the context of SD, which occurs throughout the course of aSAH but most frequently in the early phase.^116,119^ In the early to delayed phase, SD ignites in response to wide ranges of injurious stimuli, including elevated extracellular potassium ([K^+^]_o_), NO/ET-1 imbalance, prior ischaemia and electrographic seizures.^2,46^ Under physiological conditions, SD typically evokes a brief vasoconstriction at the outset of depolarisation, followed by strong hyperaemic response due to the immense energetic burden to recover membrane gradients.^116^ In acute brain pathologies such as aSAH, the initial vasoconstriction becomes amplified while the subsequent hyperaemic response diminishes, leading to spreading ischaemia that propagates with the wavefront of neural cell depolarisation (no longer following vascular territories).^120^ Such localised blood flow disruption secondary to SD is now well-documented in post-neurosurgical aSAH patients thanks to multimodal subdural electrodes with integrated flowmetry.^119,121^ Importantly, inverse neurovascular coupling impairs full recovery from SD, resulting in a positive feedback loop of self-reinforcing prolonged depolarisations and worsening vasoconstriction that develop into focal cerebral ischaemia and infarctions. The inversion of neurovascular coupling is additive to the effects of previously discussed constrictor-dilator imbalance and smooth muscle dysfunction, altogether enhancing the tissue propensity for pathological vasoconstriction. See Supplementary Material for theoretical mechanisms underlying this vascular phenomenon.

### The evolving microvascular thrombosis and capillary dysfunction

Intraluminal obstruction by diffuse plugging of small arterioles, venules and capillaries by microthrombi represents another threat to cerebral perfusion from early to delayed phase. Brain regions with the highest microthrombi load are closely associated with focal anaemic infarcts.^122^ Further, the distribution of microthrombi poorly overlaps with territories of spastic large arteries but correlates well with constricted small arterioles, suggesting microvascular thrombosis is an integral non-vasospastic component of microcirculatory dysfunction.^123^ As discussed previously, microvascular thrombosis initiates rapidly in the acute phase and develops into either transient platelet aggregates or true microthrombi, the latter of which may persist well into the delayed phase. Due to the lack of definitive methods to detect microthrombi in vivo, the current understanding on the spatiotemporal distribution of cerebral thrombotic events is still largely derived from animal models and a limited number of human autopsy studies. Nevertheless, several promising thrombus-specific molecular imaging strategies are currently under development in ischaemic stroke and venous thromboembolism research, which could be adapted for aSAH patients in the near future (see Supplementary Material).

In animal models, formation of platelet aggregates follows a biphasic pattern but typically dissipates completely in the early phase (by 48 h).^37^ Progression of microvascular thrombosis is similarly biphasic, first peaking at 48–72 h and then from day 7 onwards, coinciding with both the transition period from early to delayed phase and peak incidence of DCI.^124^ Across human and animal studies, the association between microvascular thrombosis and cerebral ischaemia-infarction is well established.^125,126^ However, the underlying mechanisms remain incompletely understood. In this section we will broadly discuss two interconnected pathways of microthrombi formation: either canonical thrombosis occurring through classical platelet aggregation and fibrinogen deposition, or non-canonical thrombosis dependent on cerebral neutrophil extracellular traps (NET).

### Systemic hypercoagulability promotes thrombosis

Haemostasis begins immediately following aneurysmal rupture and the plugging of a haemorrhaging vessel completes within minutes in the absence of overt coagulopathy.^37^ However, a hypercoagulable state can develop and persist in a subset of aSAH patients, directly contributing to microvascular thrombosis and downstream brain injuries.^127–129^ The presence of systemic hypercoagulability has long been hypothesised as one of the major causes of cerebral ischaemia and infarction following aSAH. However, detecting such hypercoagulable state is clinically challenging with routine laboratory assays, and its nature therefore remains elusive.

The emergence of whole-blood viscoelastic assays, such as rotational thromboelastometry (ROTEM) and thromboelastography (TEG), provides a closer approximation of in vivo clot formation, which allows more consistent characterisation of post-aSAH coagulation dynamics. Hypercoagulability develops gradually and usually becomes detectable in the early phase by day 2 and peaks in the delayed phase around day 10 post-haemorrhage.^130–132^ These temporal dynamics closely overlap with the onset and progression of microvascular thrombosis in autopsy studies.^125,133^ In general, an increased clot firmness (maximum clot firmness (MCF) in ROTEM and maximum amplitude (MA) in TEG) is the most reproducible finding in these studies, which reflects summed contribution of platelet function, fibrinogen concentration and the extent of platelet–fibrin cross-linking.^130,131,134,135^ Heightened clot firmness is most readily observed in EXTEM and FIBTEM assays in ROTEM studies, suggesting increased activities in extrinsic (tissue factor) pathways and enhanced fibrin deposition.^131,134,135^ Indeed, the contribution of fibrin polymerisation to clot strength increases in the first 72 h post-haemorrhage, rising from physiological levels (~23%) to values comparable to those observed in acute trauma (~33%).^131^ These early changes in clot strength and composition are associated with poorer clinical grade on admission and worse functional outcomes as measured by the mRS at 1 and 6 months, with FIBTEM MCF from days 3 to 5 showing the strongest correlation with 6-month outcomes.^127,134,136^ Platelet activation also increases following aSAH, and the extent of such activation likely further escalates in the early phase, as elevated circulating levels of platelet-derived chemokines, thromboxane A2 and hyperactivated coated platelets are detectable in this time window.^40,137,138^ These whole blood and platelet changes reflect the progressive development of systemic thrombogenicity from the early to delayed phase.

The underlying pathophysiology of the post-haemorrhagic hypercoagulability is poorly understood, though it is useful to recognise that microvascular thrombosis is at the intersection of both local and systemic procoagulant changes. Monocytes and neutrophils may play a major role in this regard, as they are the predominant infiltrating peripheral immune cells and function as key integrators of both inflammatory and coagulation pathways – in processes now collectively termed thromboinflammation.^139^ Apart from driving neuroinflammation that promotes local tissue to enter a procoagulant state, both circulating monocytes and neutrophils can dock with activated platelets via a P-selectin-dependent interaction to form platelet–leukocyte aggregates (PLA).^140,141^ This cellular crosstalk induces tissue factor (TF) expression on leukocyte surfaces, directly promoting activation of the coagulation cascade.^142,143^ Compared to monocytes, the procoagulant role of neutrophils is more varied. Apart from TF expression, neutrophil degranulation releases serine proteases such as cathepsin G and elastase, which may directly activate coagulation factors and platelets, or inactivate endogenous anticoagulants (see below).^139,144,145^ In the cerebral microvasculature, neutrophils or neutrophil–platelet aggregates (NPA) may plug small diameter vessels and/or form neutrophil extracellular traps (NET) that independently drive microvascular thrombosis (discussed in next section). Ultimately, both leukocyte populations may either remain within the vascular lumen, where they promote endothelial injury and thrombosis, or infiltrate the perivascular space, where they exacerbate neuroinflammation and vascular remodelling.^146^

One of the major links between inflammation and systemic thrombogenicity is the imbalance between endogenous procoagulant and anticoagulant activities. Of particular importance in aSAH is dysfunction in the tissue factor–tissue factor pathway inhibitor (TF–TFPI) axis, where an increased circulating TF (either cell-bound or secreted) and decreased TFPI was observed in patients in the first 3 days post-aSAH.^132^ This imbalance likely results from tissue damage and inflammation, which upregulate TF expression both locally in injury foci and systemically via circulating PLA, coupled with inflammatory degradation of TFPI by MMPs and leukocyte derived-proteases.^147,148^ Another important anticoagulant system dysfunction in aSAH is the reduced activity of the protease ‘a disintegrin and metalloproteinase with thrombospondin motifs 13’ (ADAMTS13) following aSAH, which normally prevents pathological intravascular coagulation by cleaving hyperactive ultra-large von-Willebrand factors (vWF).^149^ Both clinical and animal studies have reported reduced ADAMTS13 activity following aSAH; supplementation of this protease in rodents further attenuated microthrombi formation and reduced DCI incidence independently of vasospasm.^38,149,150^ Indeed, a low level of ADAMTS13 in the acute and early phase is among the most promising predictive biomarkers of DCI.^151^ The cause of this reduction in ADAMTS13 activity is less clear, though likely involves a combination of inflammatory proteolytic degradation, and a relative deficiency due to increased ultra-large vWF production from injured endothelium.^149,152^ Despite promising preclinical efficacy in murine models, no human trials involving ADAMTS13 as a therapeutic target in aSAH have been published or registered at the time of writing. Nevertheless, recombinant human ADAMTS13 (apadamtase alfa) has demonstrated a favourable safety profile in phase III trials and has received regulatory approval from the U.S. Food and Drug Administration and, more recently, the Australian Register of Therapeutic Goods.^153^ Thus, ADAMTS13 supplementation represents a promising translational candidate that warrants further evaluation with small scale clinical studies.

Overall, the post-haemorrhagic thromboinflammation evolves as a result of immunothrombotic leukocyte activities, TF–TFPI imbalance and ADAMTS13 dysfunction, among others. These pathological processes combined would create the conditions for the characteristic hypercoagulability marked by heightened platelet activation and fibrin deposition after aSAH.

### Neutrophils are the main driver of non-canonical thrombosis

Neutrophils are key mediators of early neuroinflammation and are increasingly recognised as independent drivers of microvascular thrombosis in aSAH. Neutrophils are the first leukocytes to respond to aneurysmal rupture in the acute phase and their activities persist into the delayed phase.^43,154^ The spatiotemporal dynamics of neutrophil recruitment post-haemorrhage parallels the time course of cerebrovascular thrombotic events. In rodents, early infiltration begins acutely after vessel rupture and peaks around 10 min in the capillary beds.^155^ Capillary neutrophil counts then decrease towards the baseline in the first 24 h but remain elevated, followed by the second wave of infiltration around day 3.^156^ This second peak of recruitment parallels the rise of neutrophil elastase levels in DCI patients, though extra caution is required when extrapolating leukocyte behaviour across species.^157,158^ Of the recruited cells, a significant portion enter perivascular space and parenchyma, while the rest remain within vascular lumen.^155^ The compartmental distribution of infiltrating cells likely reflects their differing pathological roles, discussed in the following sections. Once activated, neutrophils may exert a variety of indirect injurious effects that disrupt microvascular blood flow, including endothelial injury through degranulation, amplification of oxidative stress, as well as NO depletion catalysed by both myeloperoxidase and neutrophil-derived ROS (see NO/ET1 axis dysfunction).^70,159^ Conversely, neutrophils can also directly occlude vascular lumina via either capillary plugging or NET-mediated thrombosis.

Capillary plugging by neutrophils stems from increased tripartite neutrophil–platelet–endothelial interactions promoted by microvascular constriction and endothelial injury in the acute phase.^160,161^ The formation of neutrophil–platelet aggregates (NPA) likely enhances this process, as non-aggregate neutrophils favour adhesion in low blood shear environments (i.e. venules), whereas NPA allows for adhesion in high blood shear conditions found in arterioles and capillaries.^162^ However, an animal study suggests the number of obstructed capillaries may be limited, and reliable methods to detect such plugging non-invasively are lacking.^161^ Therefore, direct plugging remains a hypothetical contributor to focal cerebral ischaemia in human patients, with both its occurrence and importance yet to be established.

Perhaps more relevant to microvascular thrombosis is the release of NET – a matrix of chromatin fibres and antimicrobial proteins extruded by neutrophils that normally entraps pathogenic microbes, but contributes to thrombosis in sterile inflammation. Direct observation of NET formation around microvascular territories after aSAH has been recently reported in rodent models as well as a single human brain biopsy case.^158^ Interestingly, the brain may be particularly susceptible to NET-mediated thrombosis, as even large cerebral thrombi in ischaemic stroke (retrieved by thrombectomy) exhibit greater NET content compared to their cardiac counterparts.^163^ A variety of observational studies have also explored the dynamics of NET serum biomarkers in aSAH patients, with the early phase appearing to be a critical window for change.^70,158,164–166^ For example, two highly specific NET markers, myeloperoxidase–DNA complex and citrullinated histone–DNA complex, consistently decline from days 1 to 5, whereas circulating neutrophil elastase increases from day 2 onwards.^158,166^ These changes are lagged by non-complexed citrullinated histones, which peak around day 12 in patients who developed DCI. Such pattern may reflect early phase neutrophil activation and consumption of protein-DNA complexes by thrombus adhesion, followed by degradation of NETs in the delayed phase. Nonetheless, interpretation of these circulating clinical biomarker trends remains difficult, as their levels reflect the combined effects of local NET formation and degradation, the kinetics of which is poorly characterised in the context of aSAH. At the time of writing, insights on the spatiotemporal dynamics of NET formation remain dependent on animal models.

Following recruitment to the cerebral vasculature, both luminal and perivascular neutrophils release NET, which likely promotes thrombosis through different mechanisms. In rodents, intravascular NET (iNET) develops in a biphasic manner, reaching peaks at day 1 and later at day 7.^158^ This temporal distribution is significant in that it parallels the bimodal development of peak microthrombi burden found in human autopsies.^167^ Presence of iNET in vascular lumina potently induces thrombus formation by providing both the stimulus and the scaffolding for clotting.^168^ The exposed histones bound in chromatin fibres strongly activate platelet aggregation in the NET meshwork, while the matrix of chromatin fibres further traps red blood cells, vWF and fibrinogen. Altogether, NET can sustain a stable clot independent from canonical coagulation cascades, and the resulting thrombus is thus resistant to tissue-plasminogen activator (tPA)-mediated thrombolysis.^169^ Small vessels with diameter less than 15 µm can become completely occluded, whereas larger vessels remain partially patent but suffer from additional inflammatory wall damage mediated by iNET.^158^ In the meantime, perivascular NET (pNET) formation also rises in days 1–2.^165^ The role of pNET in thrombosis appears less direct, mainly through enhancing neuroinflammation and microvascular spasm upstream of the capillary beds, especially at the level of pial arterioles. Segments of pial arterioles infiltrated with pNET constrict up to 40% of the original diameter and exhibit beads-on-string appearance.^165^ The resulting blood stasis and inflammatory vascular injury likely augments microthrombi formation downstream. However, the vast majority of high-resolution data are still derived from animal models, which require cautious interpretation due to interspecies differences in neutrophil biology and cerebrovascular architecture. Through inflammation, microvascular plugging and NETosis, neutrophil activity permeates the entire course of secondary brain injury evolution following aSAH. Nonetheless, many critical insights remain lacking. For example, the true burden of NET-driven microthrombi in human patients remains unclear. On the mechanistic side, the identities of the NET triggers and the cellular pathway of NETosis following aSAH are also poorly understood.

### Integrating different levels of vascular dysfunction

The impact of post-aSAH circulatory dysfunction on tissue perfusion depends upon the synergy between macro- and microcirculatory impairments, which can be appreciated through basic cerebrovascular physiology. Large artery narrowing, either from vasospasm or remodelling, reduces downstream flow and perfusion pressure, yet CBF can be relatively preserved at moderate stenosis (25%–50% reduction in diameter).^170^ Relying on large vessels alone, ischaemic levels of perfusion deficit emerge mainly after diffuse or severe narrowing of arterial lumen (50%–85% reduction).^171,172^ Since moderate stenosis represents the most common pattern in post-aSAH cerebral vasospasm, standalone large artery narrowing is insufficient to produce focal cerebral ischaemia in many cases.^173^ Crucially, autoregulatory dilatation of downstream arterioles in health preserves blood flow in response to stenosis upstream, with simulations suggesting almost complete flow compensation below 50% narrowing of the parent artery.^174^ Following aSAH, however, concurrent arteriolar vasospasm and impaired autoregulation abolish this compensatory reserve, such that even mild to moderate large artery narrowing may culminate in focal cerebral ischaemia downstream. In poor-grade patients, elevated ICP further reduces CPP at the pre-stenotic arterial segments, exacerbating CBF loss to these compounding macro- and microcirculatory perturbations. Although these effects alone can induce tissue ischaemia, their impact is amplified by downstream capillary dysfunction. The cerebral capillary bed is subject to the same inflammatory and ischaemic insults as larger vessels and exhibits analogous morphological changes, including endothelial cell corrugation, luminal membrane projections/microvilli formation and luminal compression by swelling of astrocytic end feet.^175^ Pericytes may also constrict and further increase vascular resistance at the capillary level, though their role in post-aSAH perfusion deficits remains controversial.^176,177^ These ultrastructural disruptions would further augment flow reduction and blood stasis in the microvasculature, promoting microthrombi formation in conjunction with systemic hypercoagulability and NETosis.

Capillary occlusions have further implications for the mechanism of focal cerebral ischaemia post aSAH. Optimal tissue oxygenation requires uniform RBC distribution and similar transit times across capillary branches.^178^ When capillary flow is heterogeneous due to occlusion, the occluded branches receive little RBC or O_2_, while patent branches receive higher RBC flux and must shunt blood quickly, ultimately impairing oxygen extraction in the entire capillary bed. The extent of shunting and the corresponding impairment in tissue oxygenation can be quantified by capillary transit time heterogeneity (CTH, as the standard deviation of transit times between branches). Apart from occlusion, SD propagating across the capillary territories also severely amplifies CTH by preferentially reducing RBC flow velocity in branches that already exhibit low pre-SD flow rate.^178,179^ An increased CTH can be observed in rodents following aSAH and TBI, using intra-vital microscopy.^180^ Unfortunately, there still lacks reliable and minimally invasive detection methods in human patients, though promising neuroimaging-based proxies for CTH are being actively developed, such as mean transit time (MTT) heterogeneity in CTP and high-resolution dynamic contrast enhanced MR.^181–183^ At the time of writing, only one study utilised coefficient of variation for MTT (cvMTT) as a CTH surrogate in aSAH patients, and found that increased cvMTT at any time point correlates with poorer clinical grade at presentation, high admission mFisher and overall worse mRS outcome at 6 months.^182^ An important aspect of CTH is that increasing blood flow upstream of the capillary bed paradoxically worsens tissue oxygenation and exacerbates ischaemic injuries, due to increased shunting in non-occluded branches. Therefore, an effective way to lower CTH and improve oxygen extraction is to reduce blood flow entering the capillary bed.^178^ Such flow dynamics have several interesting implications: (1) increased CTH may partially explain no-reflow phenomenon and reperfusion injuries after the restoration of CBF in the acute phase of aSAH, (2) there may exist a novel neurovascular coupling mechanism that specifically regulates CTH and (3) large artery vasospasm may potentially have an adaptive role in reducing downstream capillary CTH and improve brain oxygenation.^180^ Nevertheless, the contribution of CTH to DCI in human patients requires further validation given the limited number of clinical studies at present.

### Bioenergetic failure leads to relative ischaemia

Relative ischaemia after aSAH is primarily caused by excess neuroelectric discharges due to abnormal neuronal depolarisation or hyperexcitability, leading to local supply–demand mismatch from heightened neural metabolism, independent from vascular compromise. At the cellular level, increased metabolic demand arises against a backdrop of heightened pathological glycolysis and impaired oxidative metabolism, beginning within hours of the bleed and persisting into the delayed phase.^157,184^ At the tissue level, the effect of relative ischaemia is additive to the concurrent perfusion deficits caused by vasospasm and thrombosis, altogether contributing to focal cerebral ischaemia and subsequent infarction. Electrographic seizures and SD are the two main interconnected electrical events that frequently co-occur and predispose relative ischaemia post-haemorrhage.^185^ Seizure may occur at any time point post-aneurysmal rupture. The classification of post-haemorrhagic seizure remains inconsistent and is often loosely based on the timing of attack: onset seizure occurs in the acute phase (<24 h or symptomatic attack immediately after rupture) and is rapidly ignited by pressure effect of haemorrhage; early seizure generally refers to attacks during admission and thus spans both early and delayed phase (within 1 week); late seizure occurs following hospital discharge, probably reflecting the epileptogenic nature of aSAH-associated neurological injuries.^186–188^ Focal epileptic discharges can trigger SD directly whereas SD lowers the threshold for seizure ignition.^189^ Both phenomena thus frequently co-occur in aSAH and are mutually reinforcing; though by definition, active ictal discharges are suppressed by SD, as the latter abolishes all electrical activities in the affected cells.^189^ Nevertheless, the interaction between seizure and SD is complex and not fully understood. For example, electrographic seizure typically propagates 10 times faster than SD, yet ictal epileptic field potentials could also spread at the same speed by trailing an SD wavefront (termed ‘spreading convulsion’). Moreover, despite acute brain injuries favouring both seizure and SD, brain changes in refractory epilepsy reduce propensity for SD.^190^ These discrepancies suggest that while both phenomena favour similar initial conditions, their subsequent trajectories diverge depending on the underlying tissue injury state.

The bioenergetic burden from these electrical events is difficult to measure in vivo. In animal models, epileptic seizure leads to a three- to five-fold increase in energy consumption in healthy, well-perfused brains.^191,192^ Both epileptic discharge and SD lead to loss of thermodynamic potential stored in transmembrane ion gradients, which could be quantified with Gibbs free energy.^193^ For comparison, epileptic seizure is estimated to release 3 J/kg of tissue whereas SD releases seven-fold higher at 22 J/kg. Even in healthy brain tissue without perfusion deficits, the energy consumption for SD repolarisation outmatches capacity of oxygen delivery, to the point that Hb desaturates to ischaemic level for several minutes.^194,195^ However, in the presence of post-aSAH perfusion deficit discussed earlier, the detrimental effect of heightened metabolic demand can be appreciated through invasive microdialysis monitoring. Although the metabolic impact of seizures remains poorly characterised and appears limited,^196^ SD consistently reduces extracellular glucose and increases lactate concentrations, reflecting a shift towards anaerobic glycolysis.^197^ These metabolic disturbances can persist for tens of minutes, likely due to concurrent perfusion deficits that prevent recovery. While the effect of an individual SD is modest (−0.032 mM glucose and +0.023 mM lactate), repetitive SD clusters additively may drive metabolite concentrations beyond thresholds predictive of subsequent DCI (<0.26 mM glucose and >7 mM lactate).^198,199^ Despite its grave consequences, clinical SD monitoring remains challenging and often invasive, while SD suppression as a therapeutic strategy is still in its infancy (see Supplementary Material).

Viability of neurons and glia following these neuroelectric events depends on the restoration of ionic homeostasis; however, the summed effect of impaired perfusion coupled with neuroelectric discharges after aSAH impose sustained energetic stress that prevents full recovery, ultimately leading to necrosis or the initiation of cell death programming.^46^

## Conclusion

Despite significant scientific progress and paradigm shifts in our understanding of aSAH pathophysiology over the recent decades, several high-priority research gaps persist. First, acute phase events such as hyperacute vasospasm and early microthrombi formation are understudied, with both their clinical significance and mechanistic underpinnings remaining largely unknown. Second, the dynamics and pathological roles of post-bleed vascular remodelling are yet unclear. Third, the central mechanisms underlying systemic hypercoagulability have not been fully investigated, and its pathological sequelae remain incompletely understood. In the therapeutic space, ADAMTS13 supplementation may be a compelling translational candidate in the subset of patients that develop a post-bleed hypercoagulable state. Finally, the precise cellular pathways of NETosis following aSAH, and its contribution to focal cerebral ischaemia, remain to be elucidated.

Overall, a ‘unified theory’ that integrates the spatiotemporal evolution of neurological injury with the relative contributions and interactions of primary mechanistic drivers remains lacking. Important concepts such as EBI and DCI, as currently defined, reflect heterogenous and interacting biological processes more so than singular pathophysiological constructs. Thus, there likely exists a need to refine and rethink these terms as clinicopathological entities, towards more mechanistically driven definitions as our understandings progress. A parallel may be drawn with the evolution of myocardial infarction, which transitioned from symptom and ECG-based criteria to the modern universal definition encompassing distinct aetiological subtypes. A mechanistically integrated understanding of aSAH, enabled by multimodal monitoring and molecular biomarkers, may ultimately define distinct DCI phenotypes and pave the way for precision neuromonitoring and personalised therapies.

## Supplemental Material

sj-docx-1-jcb-10.1177_0271678X261474706 – Supplemental material for Haemorrhage, hypercoagulability and ischaemia: Evolution of brain injury after aneurysmal subarachnoid haemorrhage Supplemental material, sj-docx-1-jcb-10.1177_0271678X261474706 for Haemorrhage, hypercoagulability and ischaemia: Evolution of brain injury after aneurysmal subarachnoid haemorrhage by Yuyang Wang, Jordan Fisker, Lipi Mishra and Matthew H Anstey in Journal of Cerebral Blood Flow & Metabolism
