# Supplementary material for: Haemorrhage, hypercoagulability and ischaemia: Evolution of brain injury after aneurysmal subarachnoid haemorrhage
Source: J Cereb Blood Flow Metab. 2026 Jul 24:0271678X261474706. Online ahead of print. doi: 10.1177/0271678X261474706 (PMC13473328; doi:10.1177/0271678X261474706)
Supplement: Supplementary file 1 [file sj-docx-1-jcb-10.1177_0271678X261474706.docx]

**Mechanism of Inverse Neurovascular Coupling**

There are probably multiple mechanisms underlying the inversion of neurovascular coupling, which are not fully understood. The prior mentioned nitric oxide (NO) dysfunction in the early to delayed phase likely impairs vasodilation; in this regard, reduced nNOS activity and uncoupling may be particularly relevant to the disruption of functional hyperaemia ^1^. Additionally, downstream to NO dysfunction, the reversal of normal K^+^ dynamics in vascular smooth muscle cells appears to play an important role ^2–4^. [K^+^]_o_ derived from neural activities regulates vascular smooth muscle via the inward-rectifying potassium (Kir) channels. In health, mild elevation of [K^+^]_o_ increases Kir channel conductance, leading to K^+^ efflux, smooth muscle hyperpolarisation, and vasodilation ^5^. However, following aSAH, accumulation of excess [K^+^]_o_ (> 20 mM) in metabolically challenged tissue leads to a large positive shift of K^+^ equilibrium potential (E_K_), reversing the direction of K^+^ current through Kir channels, and resulting in smooth muscle depolarisation and paradoxical vasoconstriction ^6^. Many sources contribute to the high extracellular K^+^ following aSAH, including haemolysis of extravasated erythrocytes, K^+^ release from SD/seizure, and impaired glial spatial buffering ^7^. Hb-derivatives such as propendyopents and BOX may also stimulate a vasoconstrictive response to functional activation, through an incompletely understood BK channel-driven astrocytic release of [K^+^]_o_ ^8^.

**Emerging Methods of Microthrombi Detection**

Several promising molecular imaging strategies are currently under development in ischaemic stroke and venous thromboembolism research. The gadolinium-based EP-2104R-enhanced MR approach is the most clinically mature, having been validated in a phase II trial ^9^. This agent relies on highly specific binding between polymerised fibrin and the Gd-labelled peptide dye, thereby selectively enhancing otherwise undetectable thrombi on a standard 1.5-T MRI scanner ^10^. Dual positron emission tomography (PET)/MRI has also been proposed as an extension of this strategy, owing to the superior thrombus sensitivity of PET ^11^. Polydopamine-coated iron oxide nanoparticles represent another promising thrombus-specific MR contrast agent, which showed favourable microthrombi sensitivity in murine models of thromboembolic stroke ^12^. With increasing recognition of aSAH as an ischaemic-haemorrhagic entity, these approaches could be readily adapted for the characterisation of post-bleed microthrombi in the near future.

**Spreading Depolarisation Monitoring and Therapeutic Targeting**

Unlike electrographic seizures, the tool kit for clinical SD monitoring remains limited. Standard electroencephalography (EEG) struggles to capture the slow SD changes due to the intrinsic high-pass filtering from capacitance of dura, skull, and skin-electrode interface ^13^. Consequently, electrocorticography (ECoG) is the mainstay monitoring modality. Placement of a subdural electrode through a craniotomy burr hole provides excellent focal spatiotemporal resolution of events and can be adapted into multimodal probes incorporating flowmetry and microdialysis ^14,15^. However, ECoG is invasive and offers limited tissue coverage. MR-based techniques represent the principal non-invasive alternative, with diffusion-weighted imaging (DWI-MR) capturing the resultant cytotoxic oedema, or blood oxygen level-dependent (BOLD) imaging detecting the characteristic haemodynamic changes associated with SD ^16^. Nevertheless, MRI provides only a cross-section of SD activity at the time of study, and the feasibility of serial high-resolution MRI in the neurocritical care setting remains uncertain.

SD monitoring is not the only challenge, as there are currently no reliable strategies for suppressing SD in aSAH; even the therapeutic benefit of SD inhibition remains unclear. A variety of agents, including anti-seizure medications and migraine prophylactics, can inhibit SD in healthy, well-perfused brain tissue ^16^; however, suppressing SD in metabolically compromised tissue has proven notoriously difficult in both experimental and clinical settings ^17^. Ketamine appears promising, with continuous infusion anecdotally abolished SDs in a single case report ^18^. Additionally, the PDE III inhibitor cilostazol has shown potential in attenuating the spreading ischaemia that frequently accompanies SD ^19^. Ultimately, elucidating the pathologic manifestation, molecular mechanism, and adaptive physiological roles (if any) will expand the repertoire of translatable therapeutic targets in this space.

**Bibliography**

1.	Colonna DM, Meng W, Deal DD, et al. Neuronal NO promotes cerebral cortical hyperemia during cortical spreading depression in rabbits. *Am J Physiol-Heart Circ Physiol* 1997; 272: H1315–H1322.

2.	Sugimoto K, Chung DY. Spreading Depolarizations and Subarachnoid Hemorrhage. *Neurotherapeutics* 2020; 17: 497–510.

3.	Chang JC, Shook LL, Biag J, et al. Biphasic direct current shift, haemoglobin desaturation and neurovascular uncoupling in cortical spreading depression. *Brain* 2010; 133: 996–1012.

4.	Girouard H, Bonev AD, Hannah RM, et al. Astrocytic endfoot Ca2+ and BK channels determine both arteriolar dilation and constriction. *Proc Natl Acad Sci* 2010; 107: 3811–3816.

5.	Dunn KM, Nelson MT. Potassium Channels and Neurovascular Coupling. *Circ J Off J Jpn Circ Soc* 2010; 74: 608–616.

6.	Hinzman JM, Andaluz N, Shutter LA, et al. Inverse neurovascular coupling to cortical spreading depolarizations in severe brain trauma. *Brain* 2014; 137: 2960–2972.

7.	Antunes AP, Schiefecker AJ, Beer R, et al. Higher brain extracellular potassium is associated with brain metabolic distress and poor outcome after aneurysmal subarachnoid hemorrhage. *Crit Care* 2014; 18: R119.

8.	Dreier JP, Joerk A, Uchikawa H, et al. All Three Supersystems—Nervous, Vascular, and Immune—Contribute to the Cortical Infarcts After Subarachnoid Hemorrhage. *Transl Stroke Res* 2025; 16: 96–118.

9.	Vymazal J, Spuentrup E, Cardenas-Molina G, et al. Thrombus Imaging With Fibrin-Specific Gadolinium-Based MR Contrast Agent EP-2104R: Results of a Phase II Clinical Study of Feasibility. *Invest Radiol* 2009; 44: 697.

10.	Overoye-Chan K, Koerner S, Looby RJ, et al. EP-2104R: A Fibrin-Specific Gadolinium-Based MRI Contrast Agent for Detection of Thrombus. *J Am Chem Soc* 2008; 130: 6025–6039.

11.	Uppal R, Catana C, Ay I, et al. Bimodal Thrombus Imaging: Simultaneous PET/MR Imaging with a Fibrin-targeted Dual PET/MR Probe—Feasibility Study in Rat Model. *Radiology* 2011; 258: 812–820.

12.	Jacqmarcq C, Picot A, Flon J, et al. MRI-based microthrombi detection in stroke with polydopamine iron oxide. *Nat Commun* 2024; 15: 5070.

13.	Berhouma M, Eker OF, Dailler F, et al. Cortical Spreading Depolarizations in Aneurysmal Subarachnoid Hemorrhage: An Overview of Current Knowledge and Future Perspectives. In: Di Rocco C (ed) *Advances and Technical Standards in Neurosurgery: Volume 45*. Cham: Springer International Publishing, pp. 229–244.

14.	Dreier JP, Winkler MKL, Major S, et al. Spreading depolarizations in ischaemia after subarachnoid haemorrhage, a diagnostic phase III study. *Brain* 2022; 145: 1264–1284.

15.	Seule M, Keller E, Unterberg A, et al. The Hemodynamic Response of Spreading Depolarization Observed by Near Infrared Spectroscopy After Aneurysmal Subarachnoid Hemorrhage. *Neurocrit Care* 2015; 23: 108–112.

16.	Maldonado-McGrath D, Sacknovitz A, Aifuwa E, et al. Monitoring Cortical Spreading Depolarization: Advancements and Applications in Neurocritical Care: A Scoping Review. *Synapse* 2025; 79: e70021.

17.	Andrew RD, Hartings JA, Ayata C, et al. The Critical Role of Spreading Depolarizations in Early Brain Injury: Consensus and Contention. *Neurocrit Care* 2022; 37: 83–101.

18.	Sakowitz OW, Kiening KL, Krajewski KL, et al. Preliminary Evidence That Ketamine Inhibits Spreading Depolarizations in Acute Human Brain Injury. *Stroke* 2009; 40: e519–e522.

19.	Sugimoto K, Nomura S, Shirao S, et al. Cilostazol decreases duration of spreading depolarization and spreading ischemia after aneurysmal subarachnoid hemorrhage. *Ann Neurol* 2018; 84: 873–885.
